# Nomophobia: Is the Fear of Being without a Smartphone Associated with Problematic Use?

**DOI:** 10.3390/ijerph17176024

**Published:** 2020-08-19

**Authors:** Fareed Kaviani, Brady Robards, Kristie L. Young, Sjaan Koppel

**Affiliations:** 1 Monash Sustainable Development Institute, Monash University, Melbourne 3800, Australia; 2 School of Social Sciences, Monash University, Melbourne 3800, Australia; brady.robards@monash.edu; 3 Monash University Accident Research Centre, Monash University, Melbourne 3800, Australia; Kristie.Young@monash.edu (K.L.Y.); sjaan.koppel@monash.edu (S.K.)

**Keywords:** nomophobia, problematic mobile phone use, mobile phone dependency, dangerous mobile phone use, prohibited mobile phone use

## Abstract

Mobile phones are changing behaviour, relationships, communication and the dynamics of physical environments. As such, reliance on the device for everyday activities has increased. Consequently, “nomophobia”, defined as the fear of being without one’s mobile phone, has emerged as a new phobia. The current study aimed to determine if nomophobia can increase the likelihood of problematic dependent, prohibited and dangerous mobile phone use. The sample comprised 2838 participants (males *n* = 1337 females *n* = 1501) recruited from various online platforms. The instrument used to measure nomophobia was the Nomophobia Questionnaire (NMP-Q), while problematic mobile phone use was measured using the Problematic Mobile Phone Use Questionnaire (PMPUQ-R). The findings revealed a strong positive correlation between nomophobia and all three problematic use factors. In addition to nomophobia, regression models revealed younger age and more time spent on a mobile phone per day significantly increased problematic dependency, prohibited use and dangerous use. Males were more likely to engage in prohibited and dangerous use, while no significant gender differences were found in dependent use. These findings support the need for further research into the relationship between nomophobia and specific aspects of problematic mobile phone use, such as using a mobile phone while driving.

## 1. Introduction

Mobile phones have become essential tools of modern life [1]; globally, mobile–cellular subscriptions are 103.5 per 100 inhabitants. In Australia, where this study takes place, subscriptions are 109.6 per 100 inhabitants [2]. The ubiquity and technological evolution of the device has seen them enter and change the dynamic of environments, human behaviour and social cohesion [3]. Mobile phones have developed into the exponentially more powerful “smartphone”, internet-enabled devices affording users more features and facilities such as digital cameras, GPS navigation, media players, applications facilitating social connection, access to unlimited information, games, gambling, video, music and much more [4]. Above all, however, the smartphone is a social tool, allowing users to interact and network [5] (Please note that the terms ‘smartphone’ and ‘mobile phone’ are used interchangeably).

In Australia, 84 percent of the population reported that they accessed the internet via a mobile phone, with 79 percent reporting that they used a phone to access the internet multiple times a day [6]. This uptake has both positive and negative consequences. Communication and a sense of connectedness [7] across vast distances occur at little cost [8]. The device can facilitate a sense of belonging and social inclusion [9]. Mobile applications assist in healthy eating, physical activity or chronic disease management [10], while GPS navigation and applications that manage speed assist road users [11]. There is also considerable research into the positive and even life-saving connections afforded by mobile phones in different communities, from Indigenous communities in Australia [12] to LGBTIQ+ young people [13] through to people suffering with serious mental illness [14].

In contrast, negative consequences include unhealthy and/or harmful uses such as addictive patterns of use, anti-social use such as cyber-bullying or avoiding physical social interactions [15,16] and dangerous and risky use such as using a mobile phone while driving [17], engaging in applications to facilitate risky sexual behaviour [18], or pre-existing addictions like gambling [19,20].

In order to determine the psychological predictors of one’s cognitive and behavioural interaction with their mobile phones, Walsh and colleagues [9] developed the Mobile Phone Involvement questionnaire (MPI-Q). By measuring the frequency of mobile phone use, mobile phone involvement, self-identity and validation from others, they revealed the importance of distinguishing between frequency of use and one’s psychological involvement with the smartphone, determining that, regardless of why one is using their mobile, “frequent” or “involved” use produces dependency. They also identified the need to belong, feel connected and develop a social identity as major social psychological drivers related to mobile phone use [9,21].

Taken together, these findings are significant. If time is positively correlated with dependency, and social–psychological factors such as belonging, connectedness and social identification encourage attachment to mobile phones, being without a mobile phone should then cause feelings of disconnectedness, isolation, or psychological disquiet [22].

### 1.1. Nomophobia

Indeed, not being able to communicate and losing connectedness are part of a collection of symptoms that have come to be known as nomophobia [23]. The term, an abbreviation of “no mobile phone phobia”, refers to the discomfort, nervousness or anxiety caused by being out of contact with a mobile phone [22].

Predicated upon the MPI-Q, Yildirim and Correia [23] developed a scale for measuring the “phobia”, the Nomophobia Questionnaire (NMP-Q) (Appendix A). They used the NMP-Q to study 301 undergraduate students and revealed the existence of an “irrational” fear of not being able to communicate, losing the connectedness that mobile phones facilitate, not being able to access information and the loss of convenience. Additionally, the respondents avoided environments where their smartphones were inaccessible. This begs the questions, how would these individuals behave in environments where phone use is prohibited, and does the extent to which they avoid being without a mobile phone lead to problematic phone behaviours?

As a relatively new measurement, the classification of nomophobia is contested. Similarly, the concept of “nomophobia” is complex and worth unpacking. There exists an overlap between nomophobia and other types of phobias, behavioural addictions [5], and mental illnesses [24], leading to the terms phobia and addiction being used interchangeably to define the condition [23,25,26]. Phobias are extreme or irrational fears, causing, at worst, a strong desire to avoid the situation or at best a dreaded endurance [27]. A mobile phone can be a channel for connection, learning, belonging and inclusion. To describe the fear of being without this conduit as a phobia is reductive and pathologises mobile phone use as something to be overcome or treated; to score highly on the nomophobia scale may merely demonstrate the device’s usefulness than illustrate a pathology. However, earlier studies that sought to test this concept defined nomophobia as a “disorder of the modern world” [25], and Bragazzi and Puente [22] suggested it be added to the Diagnostic and Statistical Manual of Mental Disorders (DSM).

For instance, Lee and colleagues [24] sought to examine how nomophobia’s psychometric properties might relate to existing disorders [22]. Their study employed the NMP-Q to measure its relationship with obsessiveness among 400 college-aged participants. The results indicated higher scores of obsessiveness corresponded to higher levels of nomophobia. They also found that an obsessive individual is likely to exhibit more anxiety when their mobile phone is absent [24]. This may mean nomophobia merely indicates the existence of other psychiatric disorders [22]. Other studies attempted to identify the predictors of nomophobia [28,29]. Olivencia-Carrión and colleagues [29] surveyed 968 participants (average age = 23 years) to explore a possible relationship between temperament, personality and the development of nomophobia. Two statistically significant contributors were discovered: Cooperation (people who are socially tolerant, empathetic, helpful, and compassionate) was associated with low levels of nomophobia, while reward dependence (the tendency to respond constantly and intensely to signals of reward and show a sensitivity to threat cues) was associated with higher levels of nomophobia [29]. Argumosa-Villar and colleagues [28] researched the relationship between nomophobia and personality. They found nomophobia and extraversion to be positively correlated, while conscientiousness, emotional stability and self-esteem were negatively correlated [28]. Tams et al. [30] detailed the interdependencies between nomophobia, social threat, uncertainty and control in the prediction of stress. They revealed nomophobia leads to stress by generating feelings of being socially threatened. Such feelings manifest when individuals have low certainty about how long they must be without a phone, and low control over when they can choose to regain access.

In short, the fear of being without a mobile phone has a relationship with psychometric traits that can lead to problematic behaviour; however, there is no research detailing whether nomophobia is actually problematic. Additionally, a recent systematic literature review of over 42 studies focused on nomophobia [31] found the condition exists globally; however, no studies have explored nomophobia is Australia.

### 1.2. Problematic Mobile Phone Use

How problematic use is defined, measured and contextualised is a matter of significant debate [32]. Research suggests a number of problematic behaviours can arise from smartphone use. Users can become dependent resulting in an inability to turn off the phone, can feel a sense of loss without it, feel unable to live without it or they might be unable to resist the impulse to use the phone. Using certain functions on a phone in prohibited or forbidden areas such as libraries, the cinema, aeroplanes or spaces where silence is required has also been documented. In addition to anti-social use, dangerous use of smartphones has been extensively documented, such as distracted driving [33] or crossing roads [34,35]. A variety of scales exist to measure problematic use [32]; however, this research uses the Problematic Mobile Phone Use Questionnaire (PMPUQ-R) [36] given its ubiquity and reflection of contemporary developments in mobile technologies and society. The PMPUQ-R captures three pertinent problematic themes: dependent use, prohibited use and dangerous use (Appendix B). Each factor within the scale is devised in consideration of specific criteria and rigorous research to distinguish problematic use from mere overuse or benign phone behaviours.

### 1.3. Aims and Hypothesis

The aim of the current study is to explore the relationship between nomophobia and problematic smartphone use to determine if the fear of being without one’s phone can predict problematic behaviour such as dangerous, prohibited and dependent use. This is important as studies avoid characterising smartphone overuse or dependency as an addiction due to the absence of physical health decrements. Similarly, problematic use may contribute to the same psychopathological conditions that beget nomophobia, such as feelings of isolation, low self-esteem, sensitivity to threat cues and stress. In determining whether problematic behaviours arise in the context of nomophobia, arguments can be made and interventions designed in support of reducing phone use to create better environments and improve the well-being of vulnerable phone users. It was hypothesised that individuals with higher levels of nomophobia would engage in more problematic mobile phone use.

### 1.4. Method

#### 1.4.1. Participants

Participants completed an online survey (SUDS) that took approximately 20 min to complete (described below). The survey was available online from June 2019 to August 2019. A total of 3806 participants commenced the online survey; however, 624 did not progress further than the eligibility questions and were excluded from the analyses. Three hundred and forty-four participants were also excluded because they had completed less than 51 percent of the survey, meaning they did not complete both the NMP-Q and PMPUQ-R questionnaires. The number of participants included in the current analyses was 2838. Participants comprised smartphone users over 18 with a valid Victorian driver licence and who reported that they were regular drivers (i.e., driven at least once per week during the previous month).

#### 1.4.2. Data Collection

This study formed part of a larger project examining factors that relate to smartphone use while driving. The online survey named the Smartphone Use and Driving Survey (SUDS) consisted of seven sections administered in the following order: (a) socio-demographic characteristics (e.g., age, gender, income), (b) average time spent daily using a smartphone, (c) nomophobia severity (NMP-Q), (d) problematic mobile phone use (PMPUQ-R), (e) driving and smartphone use history, (f) formal and informal deterrence effects and (g) smartphone use while driving behaviours. This study, however, focussed on the first four measurements: socio-demographic characteristics, daily time spent using a smartphone, nomophobia severity and problematic mobile phone use.

##### Socio-Demographic Characteristics and Smartphone Use

Participants were asked to provide information about their age group, gender, combined household income (before tax) and highest degree or level of education completed. Participants were also asked about their smartphone (i.e., whether they were Android or iPhone users). Daily time spent using a smartphone use was measured by asking participants how many hours per day, on average, they spent using their smartphone. Instructions for acquiring a real-time reading were provided to participants for both Android and iPhone users by directing them to their phone’s inbuilt screen-time application.

##### Nomophobia Severity

Nomophobia severity, or participants’ psychological attachment to their mobile phone, was measured using the NMP-Q [23] (Appendix A). The NMP-Q is a 20-item questionnaire, where each item is rated on a 7-point Likert scale (where 1 = Strongly disagree, 7 = Strongly agree). The NMP-Q comprises four factors; (1) not being able to communicate, (2) losing connectedness, (3) not being able to access information and (4) giving up convenience [23]. Nomophobia severity is calculated by summing item responses to produce a total score ranging from 20 to 140, where higher scores represent higher levels of nomophobia. As per Yildirim and Correia’s [23] scoring instructions, an NMP-Q score less than or equal to 20 indicates the “absence” of nomophobia; an NMP-Q score greater than 20 and less than 60 corresponds to a “mild” level of nomophobia; an NMP-Q score greater than 60 and less than 100 corresponds to a moderate level of nomophobia; and an NMP-Q score greater than or equal to 100 corresponds to a severe level of nomophobia. The NMP-Q has excellent internal consistency for all the items (Cronbach’s alpha = 0.95) (23, p. 135).

##### Problematic Mobile Phone Use

The PMPUQ-R is a 16-item questionnaire divided into three factors: dependent use, prohibited use and dangerous use [36]. Each item is rated on a 4-point Likert scale (where 1 = Strongly disagree, 4 = Strongly agree). Overall scores ranged from 16 to 64, where higher scores represent a greater likelihood of potential problems arising due to mobile phone use. The PMPUQ-R also has excellent internal consistency for all the items (Cronbach’s alpha = 0.86) [36].

### 1.5. Procedure

Ethics approval for this study was granted by the Monash University Human Research Ethics Committee. Participants were invited to complete the online survey on a voluntary basis through various online avenues. The survey was advertised on the VicRoads Facebook page, among the Community Road Safety Councils, the Royal Automotive Club of Victoria’s (RACV) newsletter, Monash University’s student Facebook page and a Facebook page created especially for the survey. All posts explained the purpose of the study and that participants who completed the survey could opt to go into a draw to win one of four $100 gift-card prizes.

### 1.6. Data Analysis

Descriptive analyses were conducted to reveal participant demographics (Table 1) and daily smartphone use (Table 2). Chi-square tests were conducted to explore the relationships between socio-demographic characteristics and daily time spent using a smartphone (Table 3). Nomophobia severity was described (Table 4), and chi-square tests explored the relationship between socio-demographic characteristics and nomophobia severity levels (Table 5).

Three separate regression models were conducted to explore the impact of age, gender, average hours spent per day on smartphone and nomophobia severity as predictors of each problematic mobile phone use factor (dependent, prohibited, dangerous).

Scores for dependent use were normally distributed, and therefore, a multiple regression model was conducted. However, the scores for prohibited and dangerous use were not normally distributed (i.e., positively skewed by over 30 percent) and were therefore transformed into binary variables (where 0 = no problematic use, and 1 = problematic use), and logistic regression models were conducted. All statistical analyses were conducted using IBM SPSS v 25 (IBM SPSS, Inc., Chicago, IL, USA).

## 2. Results

### 2.1. Socio-Demographic Characteristics

As shown in Table 1, participants were most likely to be aged between 40 and 59 years (34.2 percent) and female (52.9 percent).

### 2.2. Smartphone Use

Participants were asked about their smartphone type and the number of hours per day, on average, that they spend using their smartphone. More than half of the participants (53.8 percent) reported that they used an iPhone, while the remaining participants (46.2 percent) reported using an Android. As shown in Table 2, most participants (56.7 percent) reported that they used their smartphone up to three hours per day, and the remaining reported that they used it three hours or more per day.

Chi-square tests were conducted to explore the relationships between daily time spent using a smartphone (≤3 h vs. >3 h per day) and age group and gender. As shown in Table 3, there was a significant relationship between daily time spent using a smartphone and age group. Participants aged 18–25 years were more likely to spend more than three hours on their phone per day compared to all other age groups, while participants aged 60 years and older were more likely to spend three hours or less per day on their smartphones. There was also a significant relationship between daily time spent using a smartphone per day and gender. Males were more likely to spend three hours or less per day on their smartphones compared to females.

### 2.3. Nomophobia

Participants’ responses to the nomophobia scale were also assessed. The mean nomophobia score was 69.4 (SD = 25.1, Range = 20.0–140.0) and had excellent internal consistency (Cronbach’s Alpha = 0.955). The distribution was approximately symmetric with a skewness value of 0.26. Participants’ nomophobia scores were then classified into one of four nomophobia severity categories as described by Yildirim and Correia [23]. As shown in Table 4, most participants were classified as having a “moderate” level of nomophobia (48.7 percent). It was also interesting to note that less than one percent of participants had an “absence” level of nomophobia (0.8 percent), while 13.2 percent were classified as having a “severe” level of nomophobia.

Chi-square tests were conducted to explore the relationships between age, gender, average hours spent on a smartphone per day and levels of nomophobia severity (Table 5). There were significant relationships between levels of nomophobia severity, age, gender and average hours per day spent on a smartphone. Older participants (i.e., aged 40–59 years and aged 60+ years) were less likely to experience moderate or severe levels of nomophobia, while younger participants (i.e., aged 18–25 years) were more likely to experience severe levels of nomophobia. Female participants were more likely than male participants to experience moderate or severe levels of nomophobia, while participants who spend more than three hours on their smartphone per day were more likely to experience severe levels of nomophobia compared to participants who spend three hours or less per day.

### 2.4. Relationship between Nomophobia and Problematic Mobile Phone Use

A multiple regression model was conducted to investigate the impact of age group, gender, level of nomophobia severity and hours spent using a smartphone per day on the likelihood that participants engage in dependent mobile phone use (Table 6).

The multiple regression model was statistically significant, *f*(4) = 657.307, *p* < 0.001. The model explained 48.7 percent (R^2^) of the variance in dependent mobile phone use and had great internal consistency (Cronbach’s Alpha = 0.86). All degrees of nomophobia were significant predictors of dependent use. That is, the greater the nomophobia, the more likely problematic dependent behaviours occur. Those scoring severe levels of nomophobia were 11.7 times more likely than the absence of nomophobia cohort to be problematically dependent on their mobile phones. Age was strongly negatively correlated with participants’ problematic dependent mobile phone use, meaning younger phone users were more likely to be dependent. Spending over three hours a day on a smartphone was a significant predictor of greater dependent use. Gender did not significantly predict participants’ dependent mobile phone use.

Logistic regression models were conducted to investigate the impact of age group, gender, level of nomophobia severity and hours spent using a mobile phone per day on the likelihood that participants engage in prohibited mobile phone use (Yes, No) or dangerous mobile phone use (Yes, No) (Table 7 and Table 8).

The logistic regression model was statistically significant, χ^2^(8) = 555.214, *p* < 0.001. The model explained 18.1 percent (Cox and Snell R^2^) to 25.4 percent (Nagelkerke R^2^) of the variance in prohibited mobile phone use and correctly classified 73.2 percent of cases. The prohibited factor had good internal consistency (Cronbach’s Alpha = 0.73). Younger participants were most likely to engage in prohibited mobile phone use. For example, compared to participants aged 18–25 years, participants aged 26–39 years were 54.1 percent less likely to engage in prohibited mobile phone use, while participants aged 60 years and older were 91 percent less likely to engage in prohibited mobile phone use. Male participants were 1.4 times more likely to engage in prohibited mobile phone use compared to female participants. Participants with “severe” nomophobia levels were 10.3 times more likely to engage in prohibited mobile phone use compared to participants with no nomophobia (i.e., “absence”). However, there was no significant difference in prohibited mobile phone use between participants with an absence of nomophobia or a “mild” level of nomophobia. Participants who spend over three hours per day on their mobile phones were 1.5 times more likely to engage in prohibited mobile phone use than participants who spend three hours or less per day on their mobile phones.

The logistic regression model was statistically significant, χ^2^(8) = 328.854, *p* < 0.001. The model explained 11.2 percent (Cox and Snell R^2^) to 16.0 percent (Nagelkerke R^2^) of the variance in dangerous mobile phone use and correctly classified 73.1 percent of cases and had good internal consistency (Cronbach’s Alpha = 0.71). Compared to participants aged 18–25 years, participants aged 26–39 years were 38 percent less likely to engage in dangerous mobile phone use, while participants aged 60 years and older were 70 percent less likely to engage in dangerous mobile phone use. Male participants were 1.9 times more likely to engage in dangerous mobile phone use compared to female participants. Participants with severe nomophobia were 14 times more likely to engage in dangerous mobile phone use compared to participants with no nomophobia. Participants that spend over three hours per day on their mobile phones were 1.6 times more likely to engage in dangerous use than participants that spend less than 3 h per day on their mobile phones.

## 3. Discussion

This is the first paper to measure levels of nomophobia among an Australian sample. The study explored the relationship between nomophobia severity [23] and problematic mobile phone use [36], allowing a better understanding of how an over-reliance on mobile phones may result in problematic mobile phone use such as dependent use, prohibited use and dangerous use. In the following sections, we closely examine the relationship between nomophobia and each factor of problematic smartphone use.

### 3.1. Nomophobia

Research has found that nomophobia is an emerging problem of the modern era [37]. According to the scale developed by Yildirim and Correia [23], almost all of our respondents reported some form of nomophobia; 0.8 percent reported no nomophobia (<20), 37.3 percent had a mild (21–59) level, 48.7 percent had a moderate (60–99) level and 13.2 percent reported a severe (100–140) level of nomophobia.

Although problematic mobile phone use has been explored throughout Australia [38], our study is the first to explore nomophobia among an Australian sample. This study consists of the second largest sample size (*n* = 2838) in any nomophobia study to date [31], revealing a mean nomophobia score of 69.4 (moderate) among respondents. By comparison, a sample of over 3216 Iranian adolescents showed a mean nomophobia score of 74.65 [39].

The more time spent on one’s phone was significantly associated with higher levels of nomophobia across all demographics, meaning we become more fearful of being without our mobile phones the more we use them. The relationship between nomophobia severity and several demographic factors (i.e., age group, gender, income and education) revealed that income and education are not significantly related to nomophobia levels. Some research [28,40] has found that nomophobia exists across all age groups; however, this study found that nomophobia severity decreased with age, a finding similar to other research [22].

The current study found no relationship between gender and nomophobia levels. Yildirim et al. [40] found that females had higher levels of nomophobia, while Argumosa-Villar et al. [28] and Dixit et al. [37] found no difference. These mixed results indicate gender differences may be influenced by culture, as these respective studies were conducted in Turkey, Spain and India.

Indeed, the role of culture in influencing nomophobia has been explored and established, suggesting cultural context may significantly shape differences in technology behaviours [41]. That said, what are the implications of this when our study found 99.2 percent of participants reported some form of nomophobia? Here, we find the concept of nomophobia—and the associated scales of measuring the phenomenon developed by Yildirim and Correia [23]—useful as a starting point to think about the complicated relationship individuals may have with their devices. We would not advocate for a fear of being without social connections, a sense of community or knowledge (as mediated through mobile internet technology) to be conceptualised as a disorder. Nomophobia in and of itself may not be inherently problematic; however, when the fear of being without a mobile phone produces clear and pressing implications, there arises the need to better understand and address nomophobia as a complex socio-technical phenomenon. Our research question, then, asked whether nomophobia increases the likelihood one’s mobile phone use will translate into problematic use. Additionally, it is important to consider if higher levels of nomophobia translate into higher levels of risk, and if the reported psychological characteristics of this vulnerable group, such as obsessiveness [24], low social tolerance, reward dependency and sensitivity to threat cues [29], extraversion, low self-esteem or emotional stability [28], might affect their capability to mitigate potential harms.

### 3.2. Nomophobia and Problematic Mobile Phone Use

The results of this study indicate nomophobia is a significant predictor of the likelihood one will find themselves engaging in problematic mobile phone use [36]. Research into nomophobia has tended to focus on its relationship with psychological conditions or traits, while classifications of the phenomenon tend to be numerous and overlapping [24,28,30,42]. The lack of evidence showing impaired *physical* health has contributed to obstinacy in characterising some mobile phone use as an addiction [43]. Our results provide evidence for the notion that exhibiting a fear of being without one’s mobile phone can lead to problematic dependent, prohibited or dangerous use, each factor of which may pose significant health risks, such as over-use, anti-social use or reckless and physically compromising use.

#### 3.2.1. Nomophobia and Problematic Dependent Use

Our research shows nomophobia can predict problematic mobile phone dependency. In other words, the fear of being without one’s smartphone for access to information, convenience, to maintain an online social identity and a sense of connection and belonging can increase the chance of problematic dependency by over 11 times. This finding is significant given that the results also show spending more than three hours per day on a mobile phone only increases the likelihood of problematic dependency by a third. Although a significant and large number by its own right, compared with nomophobia, it can be argued that one’s psychological relationship with their mobile phone, independent of time spent on the device per day, can greatly predict problematic dependent use.

Although some research has shown differences in mobile phone use between men and women, we found that while women were more likely to spend more time on their phones, gender was not a significant predictor of problematic dependent use. This is consistent with other research [44]. We did, however, find that younger respondents (18–25) tended to report higher levels of problematic dependent use than older age groups (60+). Other research [38] has also found age to be significant in determining problematic mobile phone use, with the 18–25 year-old age group at highest at risk. While young people have been shown to be enthusiastic adopters of new technologies [45], they are also more susceptible to dependency [19]. Although the higher prevalence of smartphone dependency among young people is known, our research shows nomophobia increases the likelihood of experiencing the problem.

Because nomophobia predicts a higher likelihood of problematic dependency, it is important to understand how the psychological predictors of nomophobia may contribute to problematic dependency, and how these less stable and desirable characteristics could in turn exacerbate dependency.

The determinants of problematic dependency—being afraid to turn off or be without one’s mobile, feeling lost or irritated without it or spending excessive time on the device—could lead to a scenario where one’s digital world takes precedence to the detriment of their physical reality. As Olivencia-Carrión et al.’s [29] study showed, those low in nomophobia have higher levels of social tolerance, perhaps gaining a sense of belonging, connectedness and identity from offline space. Becoming dependent on the sense of belonging, connectedness or social identity that smartphones can offer may inadvertently reduce one’s capacity to develop the social tools needed to nurture and navigate these bonds offline. Indeed, this dependency has been shown to act as a digital blanket [46], where the mobile phone becomes a security blanket to ameliorate social stress/exclusion. Becoming dependent on a mobile phone to reduce psychological distress becomes problematic, in that it increases nomophobia and therefore problematic dependency, becoming a barrier to psycho-social development.

Similarly, having a sensitivity to social threat [29] or being an extrovert [28] may foster a dependency on being perpetually available [47] to maintain a sense of belonging and connectedness via an online, cyber social self [48].

Olivencia-Carrión et al. [29] show nomophobia is positively correlated with reward dependence, meaning users respond constantly and intensely to signals of reward. Our research shows how this dependency also leads to problematic dependency. The balance between online and offline appears to distinguish the problematic component. Indeed, the fear of being without a mobile phone may merely be the fear of being with society. Decreasing problematic dependency by overcoming the fear of being without a mobile phone may have positive social and psychological benefits. Nonetheless, the rise in digital applications designed to alleviate stress and anxiety, increase physical and psychological well-being, moderate addictions or reduce time spent on devices—*the antecedents of dependent phone use*—may actually serve to increase dependency.

#### 3.2.2. Nomophobia and Problematic Prohibited Use

Our research shows nomophobia can also predict problematic prohibited mobile phone use. That is, the fear of being without a mobile phone to access information, convenience, maintain a sense of belonging, connection and social identity can increase the likelihood one will use a mobile phone in spaces where it is forbidden to do so such as the library, classroom, cinema or where people need silence by 10.3 times. Males were more likely to engage in prohibited use, as well as young people.

Social spaces are no longer predicated by the physical, embodied self. Social interaction also exists in a digital, disembodied form, meditated by mobile technologies [49]. These digital lives are perpetual [50], creating a context in which people are always present via their mobile phones even when not actively participating [51], creating a balancing act between the digital and physical self, and the social mores guiding each space. Prohibiting the use of a mobile phone disconnects users from their digital lives, giving precedence to their physical environment.

The fear of being without one’s mobile phone is increased by the uncertainty of knowing when one may regain access to their device [30]. Indeed, the social threat associated with being unavailable to respond, unreachable, or maintain a digital identity leads to stress [30]. Additionally, the absence of digital psycho-social rewards [29], coupled with other psychological traits such as less empathy, compassion and social tolerance, may create a scenario where the prohibited space becomes persecutory. The threat of digital social exclusion offsets the threat of physical censuring.

Indeed, prohibited use requires greater risk taking, which males [52] and younger people [53] are more inclined to do. Young people have grown up with digital technology and established their own set of rules and social norms around use that may challenge historically digital free environments. This friction between digital and physical worlds, however, has encouraged environments to adapt; in some previously prohibited spaces, such as cinemas, the device becomes permissible [54], while in others, such as libraries, digital technology is avidly incorporated [55]. In educational settings, for instance, some jurisdictions are taking to banning mobile phones from classrooms to avoid distractions and ‘cyberbullying’, and to promote in-person social interaction [56] despite education researchers arguing for more measured approaches that recognise the value of mobile phones in these settings [15,16].

#### 3.2.3. Nomophobia and Problematic Dangerous Use

Using a smartphone while driving [33], crossing the road [34,35], walking [57] or other situations that are considered dangerous is well documented. Often, predictors for smartphone use in these scenarios focus on psychological traits, risk analysis or one’s beliefs regarding attitudes, subjective norms or perceived behavioural control regarding the behaviour. Our study demonstrates the fear of being without convenient access to information, maintaining communication, connectedness and belonging can predict the likelihood one will engage in dangerous smartphone use. Specifically, the likelihood one will engage in dangerous smartphone behaviours can increase by up to 14 times for people with severe nomophobia.

Mitigating dangerous phone use has presented significant challenges in multiple arenas as outlined; however, no research has explored nomophobia as a predictor of distraction driving, crossing roads or dangerous use in general. This is important, as alongside these pervasive dangerous behaviours are emerging mobile phone applications designed to prevent such dangerous use [58]. Additionally, many psychometric predictors of nomophobia have been explored in relation to illegal smartphone use while driving, such as obsessiveness [59], extraversion [60], self-esteem [61] and the social threat associated with not answering [62], but no studies have employed the NMP-Q to assess if a fear of being without a mobile phone can better predict illegal use while driving. Notably, Seiler and Kirby [63] explored social threat in relation to the tethering effect mobile communication has on the driving environment. The norms guiding online communication—the normative expectation to respond to texts, answer calls or update social media—are significant enough to demand perpetual cognitive attention, even while driving. Here, the device is merely a means to fulfil the demands of their social role; it is not so much a reductive psychological explanation as it is a conflict of cultural expectations.

Again, as our study has found, younger age groups, independent of nomophobia, are more likely to engage in dangerous use, which is supported by much of the research around use while driving, cycling and walking [60,64]. This finding could be understood by a multitude of factors, such as the group’s proclivity for taking more risk [53] and deficient risk judgement skills [65], or in the case of road safety, their sense of invincibility [66], low perceptions around the certainty of apprehension [67,68] or engagement with compensatory driving or phone behaviours [69].

Additionally, males were almost twice as likely than females to engage in dangerous use. This is concurrent with the literature on males and their higher willingness to take risks than females [52], and the significant negative correlation between their behavioural inhibition system and problematic mobile phone use [70]. Although we do not know what phone functions males are engaging with during dangerous scenarios, research shows males typically use their phones more for task-related purposes than the social reasons typical to females [60,71].

## 4. Implications

Living in the modern world without a mobile phone can put one at a disadvantage; for many, it is not merely a matter of convenience. Sociologically speaking, the desire to seek connection in an increasingly individualised world [72], to develop a sense of belonging among a fragmentized and global community [73], to have access to unlimited information in a society that values and preferences knowledge [74] and to enjoy the instant gratification associated with convenience [75] are not, ostensibly, a collection of irrational motivations. Indeed, those most likely to be high in nomophobia embody some psychological traits—low self-esteem, emotional instability, low social tolerance—that have been shown to benefit from connection and belonging [76,77]. The increase in problematic use among higher levels of nomophobia may merely be an indicator of environmental shortcomings, where one’s offline world is deficient in the benefits a mobile phone usually facilitates. Shifting the focus from an individual’s relationship with their phone toward the substance and dynamic of their offline realities may help to inform thoughtful countermeasures geared toward enriching the absence of mobile technology rather than integrating and encouraging more use. What this may look like is a matter for policy makers; however, by defining and amplifying the rewards of offline spaces, the desire for knowledge, belonging, convenience and connection may transmute into the distinctly analogue experience of wisdom, being, discovery and connecting. This would require further empirical exploration; however, reducing problematic use may first require reducing the drivers of nomophobia.

The contribution of these findings may inform interventions aimed at reducing dangerous, prohibited and problematic dependent use. From a practical sense, it is important to consider ways to deter or accommodate vulnerable phone users from entering environments where phone use is prohibited or dangerous, or from developing habits that lead to dependency. This study has contributed to understanding the prevalence and characteristics of nomophobia within a large Australian sample, and its relationship with problematic use, which may contribute to developing better solutions to safely integrate or mitigate problematic use. As we argue, exhibiting problematic behaviours may indicate an over-reliance on one’s mobile phone to facilitate or elicit that which is lacking offline. It is not merely sufficient to reproach the individual or their behaviour—interventions must consider the existence of and reasons for nomophobia and how it may be encouraging problematic use. This might translate into campaigns that, rather than moralising problematic phone use, acknowledge the device’s important social functions and offer mechanisms for reducing problematic behaviours without disconnecting people from their network. The rate at which younger people adopt smartphone technology makes it increasingly important for parents, educators, government policy makers and the community to be aware of problematic behaviours that attachment to the device can cause, while correctly identifying all vulnerable phone users and redressing their behaviour appropriately. These results also support the need for further research into the effects of nomophobia on specific problematic environments. For instance, although a plethora of research around mobile phone driver distraction exists [78,79,80,81,82], no research has analysed dependency as a predictor for using a mobile phone illegally while driving. If people scoring high in nomophobia are more likely to use their phones illegally while driving, road safety policy could address phone habits alongside current risk-focused [83] interventions. Similarly, pre-existing psychological conditions can exacerbate nomophobia and, as our research has shown, can lead to problematic use. Addressing these antecedent traits may have a flow on effect. For instance, mindful practices have been shown to reduce problematic dangerous mobile phone behaviours within the road safety arena [84]. Such techniques may be effectively employed earlier to reduce problematic use in other environments.

## 5. Limitations

There are several limitations to this study. First, the use of self-report data is subject to bias [85]. Some participants may have overestimated or underestimated their answers to the NMP-Q or PMPUQ-R, affecting the accuracy of results. In future research, this could be mitigated by comparing results with similar studies to observe any skewed results or by conducting longitudinal studies to gain a richer understanding of problematic use among people high in nomophobia over time. Second, the PMPUQ-R may benefit from further revision, as some respondents may not consider, or know, that specific types of use are dangerous or prohibited. Additionally, any revisions should address the ambiguities of some questions. For instance, merely asking about phone use while driving does not consider the types of phone use that may or may not be dangerous, such as using the phone in a legal manner to change music while driving, or, in the case of prohibited use, using a phone in the cinema or library with permission. As such, the PMPUQ-R may not be an appropriate scale for measuring the complex reality of the dangers surrounding mobile phone use while driving. Finally, the findings from the current study are based on a convenience sample and may be the result of a volunteer bias (i.e., individuals who agreed to participate in the online survey may be more interested in smartphones or technologies generally) and who were largely recruited through social media platforms and therefore may not be representative of the general Australian population.

## 6. Conclusions

This study was the first to show the existence of nomophobia in an Australia sample. The findings of this study also show that nomophobia leads to problematic mobile phone behaviours. Additionally, higher screen time can also predict dangerous, prohibited and dependent use. We suggest future research might consider shifting the focus from an individual’s relationship with their phone toward the substance and dynamic of their offline realities. Additionally, research should determine whether nomophobia is a predictor of specific problematic behaviours such as using a phone while driving and whether reducing nomophobia can reduce specific problematic behaviours.

## Figures and Tables

**Table 1:** Socio-demographic characteristics.

Demographics	%	*n*
Gender		
Male	47.1	1337
Female	52.9	1501
Age group (years)		
18–25	16.8	478
26–39	21.7	617
40–59	34.2	971
60+	27.2	772

**Table 2:** Average number of hours spent per day using a smartphone.

Hours	%	*n*
<1	14.5	402
1–2	23.4	651
2–3	18.9	524
3–4	15.5	430
4–5	11.1	308
5–6	6.9	192
6–7	3.7	103
7–8	1.8	50
8–9	1.1	31
9+	3.2	88
Total	100.0	2779

Sixty cases excluded due to missing data.

**Table 3:** Between age, gender and average hours spent using a smartphone per day.

Demographic Characteristics		Average hours Spent Using a Smartphone Per Day		Sig.
		≤3 h % (*n*)	>3 h % (*n*)	
Age group (years)	18–25	21.7% (102)	78.3% (367)	X^2^(3) = 523.95, *p* < 0.001, ϕ*c* = 0.43
	26–39	41.0% (247)	59.0% (356)	
	40–59	63.1% (598)	36.9% (350)	
	60+	83.0% (630)	17.0% (129)	
Gender	Male	63.4% (847)	35.6% (466)	X^2^(1) = 61.09, *p* < 0.001, ϕ*c* = 0.14
	Female	49.9% (730)	50.1% (736)	

**Table 4:** Nomophobia severity level.

Severity	%	*n*
Absence (≤20)	0.8	22
Mild (21–59)	37.3	1059
Moderate (60–99)	48.7	1381
Severe (100–140)	13.2	375
Total	100.0	2837

**Table 5:** Between age, gender and average hours spent using a smartphone per day and nomophobia severity levels.

Characteristic		Nomophobia% (*n*)				Sig.
		Absence	Mild	Moderate	Severe	
Age group (years)	18–25	0.0% (0)	17.2% (82)	56.9% (272)	25.9% (124)	X^2^(9) = 287.07, *p* < 0.001, ϕ*c* = 0.184
	26–39	0.0% (0)	26.9% (166)	54.2% (334)	18.8% (116)	
	40–59	1.0% (10)	40.8% (396)	48.6% (472)	9.6% (93)	
	60+	1.6% (12)	53.8% (415)	39.2% (303)	5.4% (42)	
Gender	Male	1.2% (16)	44.1% (589)	45% (601)	9.8% (131)	X^2^(3) = 66.02, *p* < 0.001, ϕ*c* = 0.153
	Female	0.4% (6)	31.3% (470)	52.0% (780)	16.3% (244)	
Avg. hours spent per day on smartphone	<3	1.1% (18)	47.5% (749)	44.4% (699)	7.0% (110)	X^2^(3) = 227.35, *p* < 0.001, ϕ*c* = 0.286
	≥3	0.3% (4)	24% (288)	54.4% (654)	21.3% (256)	

**Table 6:** Regression predicting participants’ dependent mobile phone use.

Variable		B	S.E.	Wald	df	*p*
Age group	18–25	0	-	-	-	-
	26–39	0.52	0.20	6.74	1	0.009
	40–59	0.27	0.19	2.07	1	0.150
	60+	−0.46	0.21	4.51	1	0.034
Gender	Male	0	-	-	-	-
	Female	−0.14	0.13	1.08	1	0.300
Nomophobia severity	Absence	0	-	-	-	-
	Mild	3.52	0.69	25.69	1	0.001
	Moderate	7.71	0.69	6.34	1	0.001
	Severe	11.71	0.71	268.63	1	0.001
Avg. hours spent per day on smartphone	<3	0	-	-	-	-
	≥3	1.33	0.14	97.27	1	0.001

**Table 7:** Regression predicting likelihood of engaging in prohibited mobile phone use.

Variable		B	S.E.	Wald	df	*p*	Odds Ratio	95.0% C.I. for Odds Ratio	
								Lower	Upper
Age group	18–25	0	-	-	-	-	1	-	-
	26–39	−0.78	0.21	13.50	1	0.001	0.459	0.30	0.70
	40–59	−1.53	0.20	60.00	1	0.001	0.216	0.15	0.32
	60+	−2.41	0.20	139.00	1	0.001	0.09	0.06	0.13
Gender	Male	0.31	0.10	11.00	1	0.001	1.40	1.13	1.70
	Female	0	-	-	-	-	1	-	-
Nomophobia severity	Absence	0	-	-	-	-	1	-	-
	Mild	0.88	0.50	3.24	1	0.072	2.42	0.92	6.40
	Moderate	1.44	0.50	8.57	1	0.003	4.23	1.61	11.11
	Severe	2.33	0.52	20.22	1	0.001	10.32	3.70	28.80
Avg. hours spent per day on smartphone	<3	0	-	-	-	-	1	-	-
	≥3	0.40	0.10	12.80	1	0.001	1.50	1.22	1.82

**Table 8:** Regression predicting likelihood of engaging in dangerous problematic mobile phone use.

Variable		B	S.E.	Wald	df	*p*	Odds Ratio	95.0% C.I. for Odds Ratio	
								Lower	Upper
Age group	18–25	0	-	-	-	-	1	-	-
	26–39	−0.47	0.20	7.00	1	0.008	0.62	0.43	0.88
	40–59	−0.70	0.20	18.00	1	0.001	0.49	0.40	0.68
	60+	−1.33	0.20	57.22	-	0.001	0.30	0.20	0.37
Gender	Male	0.64	0.10	44.70	1	0.001	1.90	1.60	2.30
	Female	0	-	-	-	-	1	-	-
Nomophobia severity	Absence	0	-	-	-	-	1	-	-
	Mild	1.12	0.5	6.00	1	0.016	3.07	1.22	8.00
	Moderate	1.70	0.5	12.50	1	0.001	5.37	2.09	13.22
	Severe	2.63	0.5	27.50	1	0.001	14.00	5.21	37.41
Avg. hours spent per day on smartphone	<3	0	-	-	-	-	1	-	-
	≥3	0.50	0.10	22.00	1	0.001	1.60	1.32	2.00

**Figure A1:**
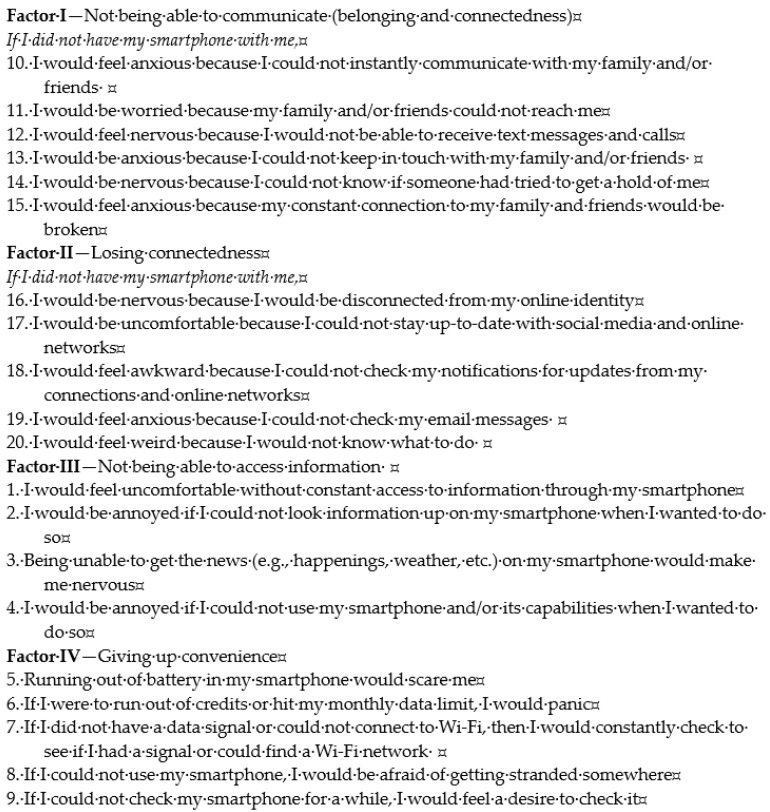
The 20 items in the Nomophobia Questionnaire (NMP-Q).

**Figure A2:**
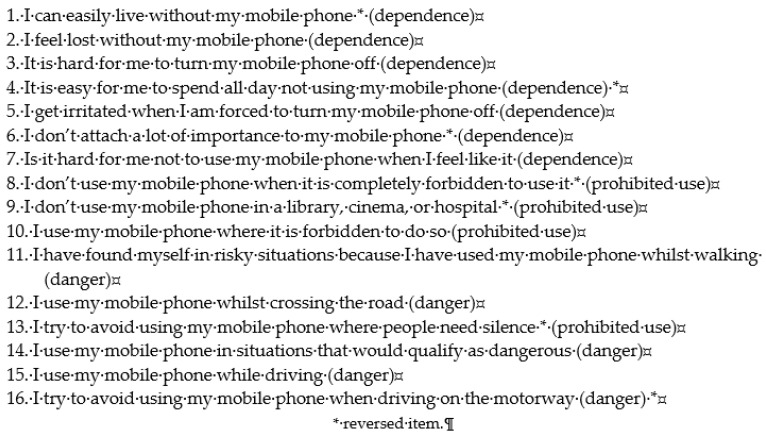
Problematic Mobile Phone Use Questionnaire (PMPUQ-R) items.
